# RAP: RNA-Seq Analysis Pipeline, a new cloud-based NGS web application

**DOI:** 10.1186/1471-2164-16-S6-S3

**Published:** 2015-06-01

**Authors:** Mattia D'Antonio, Paolo D'Onorio De Meo, Matteo Pallocca, Ernesto Picardi, Anna Maria D'Erchia, Raffaele A Calogero, Tiziana Castrignanò, Graziano Pesole

**Affiliations:** 1 CINECA - Consorzio interuniversitario per il calcolo automatico, Bologna, Italy; 2 Translational Oncogenomics Unit, Italian National Cancer Institute "Regina Elena", Rome, Italy; 3 Dipartimento di Bioscienze, Biotecnologie e Biofarmaceutica, University of Bari, Bari, Italy; 4 Dipartimento di Biotecnologie e Scienze della Salute, University of Turin, Turin, Italy; 5 Istituto di Biomembrane e Bioenergetica, Consiglio Nazionale delle Ricerche, Bari, Italy; 6 Center of Excellence in Genomics (CEGBA), Bari, Italy

**Keywords:** RNA-Seq, Alternative splicing, transcriptome regulation, workflow

## Abstract

**Background:**

The study of RNA has been dramatically improved by the introduction of Next Generation Sequencing platforms allowing massive and cheap sequencing of selected RNA fractions, also providing information on strand orientation (RNA-Seq). The complexity of transcriptomes and of their regulative pathways make RNA-Seq one of most complex field of NGS applications, addressing several aspects of the expression process (e.g. identification and quantification of expressed genes and transcripts, alternative splicing and polyadenylation, fusion genes and trans-splicing, post-transcriptional events, etc.).

Moreover, the huge volume of data generated by NGS platforms introduces unprecedented computational and technological challenges to efficiently analyze and store sequence data and results.

**Methods:**

In order to provide researchers with an effective and friendly resource for analyzing RNA-Seq data, we present here RAP (RNA-Seq Analysis Pipeline), a cloud computing web application implementing a complete but modular analysis workflow. This pipeline integrates both state-of-the-art bioinformatics tools for RNA-Seq analysis and in-house developed scripts to offer to the user a comprehensive strategy for data analysis. RAP is able to perform quality checks (adopting FastQC and NGS QC Toolkit), identify and quantify expressed genes and transcripts (with Tophat, Cufflinks and HTSeq), detect alternative splicing events (using SpliceTrap) and chimeric transcripts (with ChimeraScan). This pipeline is also able to identify splicing junctions and constitutive or alternative polyadenylation sites (implementing custom analysis modules) and call for statistically significant differences in genes and transcripts expression, splicing pattern and polyadenylation site usage (using Cuffdiff2 and DESeq).

**Results:**

Through a user friendly web interface, the RAP workflow can be suitably customized by the user and it is automatically executed on our cloud computing environment. This strategy allows to access to bioinformatics tools and computational resources without specific bioinformatics and IT skills. RAP provides a set of tabular and graphical results that can be helpful to browse, filter and export analyzed data, according to the user needs.

## Background

RNA-Seq has become one of the most popular technique across the vast landscape of the next and third generation sequencing technologies [1]. It can be profitably used to investigate the gene expression process, estimating both the nature and the quantity of expressed mRNAs [2] by sequencing a complete transcriptome in any cell/tissue type and condition. The ability to simultaneously detect and quantify the expression profile for a large number of genes in specific physiological and pathological conditions has opened new avenues for a deeper understanding of biological processes and their regulation (e.g. genome-wide investigation of epigenetic inheritance) and paved the way for several biotechnological and biomedical applications.

Indeed, RNA-Seq can identify and quantify expressed genes and transcripts providing precious biological information on the underlying gene expression mechanisms. Notably, gene expression is a highly regulated process and in some cases final products cannot be fully characterized by analyzing short reads generated by NGS platforms particularly when many alternative transcripts of remarkable length are generated due to complex co-transcriptional and post-transcriptional nuclear processing, including alternative initiation and termination of transcription and alternative splicing [3].

RNA-Seq data can be analyzed by adopting several computational strategies also depending on the requested results (e.g. expression at gene and/or transcript level, investigation of alternative splicing events, alternative polyadenylation sites, etc.). However, despite recent technological advances, key transcriptome features are yet to be fully elucidated, and its scale and complexity have not yet been fully understood [4]. In order to provide easy and effective access to the gene expression studies to researchers with few or limited bioinformatics skills, user-friendly automated workflows are highly demanded to provide reliable and easy interpretable results [5] which also keep up with the exponential growth of sequencing technologies [6].

To the authors knowledge, several pipeline tools have been implemented for RNA-seq data analysis [7-13], some of them proposed along with novel algorithmic approaches to refine final results [7,8]. However, most of them are totally lacking an easy accessible web interface for cloud computing [7-9]. Most of them do not allow to execute a complete pipeline from read mapping to advanced processing tasks since they only implement a few specific steps [11-13]. Some tools [13] are implemented as Galaxy [14] customized instances, extending the platform contribution to the bioinformatics community. Unfortunately, they do not feature a powerful responsive web application to browse, filter and explore the analysis results, that is, in our experience, the main interest of final users.

In this paper we present the implementation of a modular analysis workflow, named RAP (RNA-Seq Analysis Pipeline), designed to analyze sequencing data in multiple steps, each one addressing a specific task.

The purpose of RAP is to investigate the complex transcriptional landscape of eukaryotic transcriptomes through a computationally optimized RNA-Seq data analysis. This tool is a web application implementing an automatic but completely customizable analysis workflow able to carry out a comprehensive transcriptome analysis and provide a wide range of results, which consist of several tabular and graphical representations that can be suitably organized, filtered and browsed according to the user needs.

The workflow is presented as a multi-step pipeline. The expressed isoforms are reconstructed by adopting spliced mapping algorithms to align reads to the genome and assembling them into full-length transcripts. Then, through isoforms deconvolution methods, transcripts expression is quantified. Alternative splicing is investigated by mapping reads against an exhaustive splice junctions library constructed by a comprehensive combinatorial assortments of known splicing events. Computational strategies implemented in RAP modules also allow to identify polyadenylation sites, elementary alternative splicing events (e.g. exon skipping) and chimeric transcripts. Finally, the comparative analysis of two or more experiment groups corresponding to different physiological or pathological conditions allows the detection of statistically significant changes in expression levels at gene or transcript isoform level, in the splicing pattern (e.g. differential exon skipping) and in used polyadenylation sites.

This broad variety of different analysis branches is achieved by means of a highly modularized implementation and a fully generalized computational engine.

## Implementation

**Figure 1:**
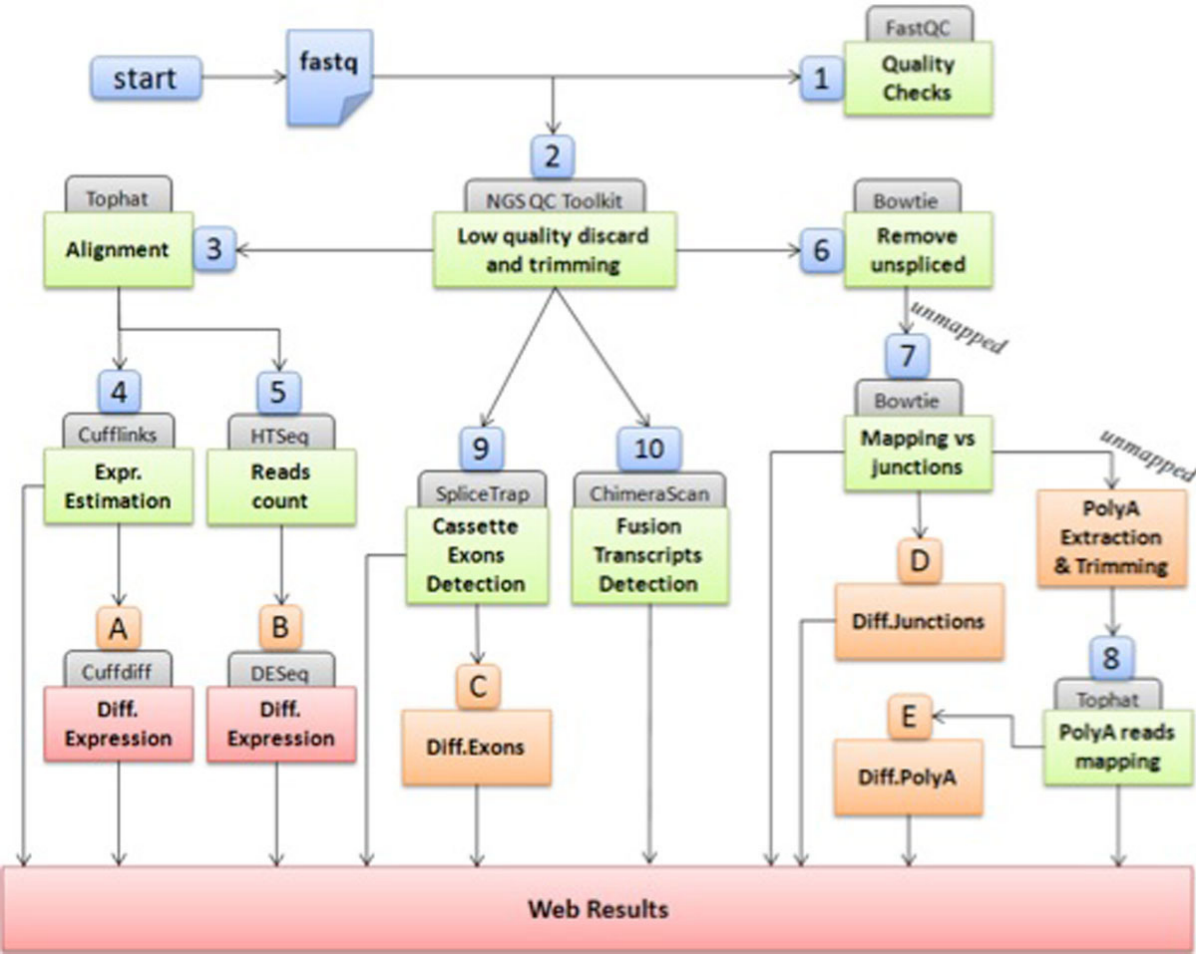
**Schematic description of RAP workflow**. Quality check and filtering step for quality assessment (Steps 1 and 2). High quality reads are aligned to the reference genome using TopHat (Step 3). Alignments are assembled into full-length transcripts and their relative abundances are estimated by Cufflinks to (Step 4) and raw-counted by HTSeq (Step 5). Unspliced reads are filtered out after an ungapped alignment to the genome (Step 6). Remaining reads (potentially spliced) are mapped to a custom built junction library (Step 7). Reads still unmapped are scanned to identify poly(A) tags (Step 8). Cassette exons are identified and quantified by adopting a statistical tool, SpliceTrap (Step 9), and chimeric transcripts are detected by means of ChimeraScan (Step 10). After the completion of the main analysis, several differential analyses can be executed. Cuffdiff at transcript level (Step A), based on expression levels calculated by Cufflinks. DESeq at gene level (Step B), based on gene raw counts calculated by HTSeq. Differential exons (Step C), differential junctions usage (Step D) and differential polyadenylation sites (Step E) can also be calculated.

RAP (RNA-Seq Analysis Pipeline) is a web application implementing a fully automated analysis workflow, designed to integrate in-house developed scripts as well as open source analysis tools into one pipeline (Figure 1). Using RAP the user can perform a complete RNA-Seq analysis without any specific technical competence nor directly managing the complexity of distributed computational resources. Moreover RAP also offers a web interface for results management and visualization, allowing the user to browse and filter the massive amount of data obtained from typical RNA-Seq experiments.

RAP takes as input short-read datasets produced by Illumina sequencing platforms and supports several standard file formats (FASTQ, SRA, BAM and compressed archives).

The pipeline is designed to analyze the data through a series of phases, each of them focused on a specific task.

RAP integrates two widespread tools for quality assessment: FastQC [15] and NGS QC Toolkit [16] both providing quality checks and statistics, useful for a preliminary evaluation and filtering of raw data. After the quality control and reads filtering phase, different paths can be simultaneously followed, according to the user's request.

The *first *path implements TopHat [17]/ Cufflinks [18] / Cuffdiff2 [19] and/or HTSeq/DESeq [20] for gene/isoform expression quantification and differential analysis.

The *second *one detects and quantifies alternative splicing events, particularly cassette exons and their differential occurrence through SpliceTrap [21].

The *third *path detects and quantifies splicing junctions, including novel ones as well as polyadenylation sites and their differential occurrence in different samples. The splice junctions library is built starting from reference gene annotation models and includes both known and potential splice junctions (see below for further details).

Residual reads still unmapped to the genome, transcriptome and junctions are analyzed to eventually detect polyadenylation sites. PolyA tags (reads containing a stretch of A (An) at the end of the sequence) are extracted, An-trimmed and re-aligned to the genome. Following the alignment, a pattern matching procedure is also applied to possibly detect common polyadenylation signal sequences (PAS) such as AAUAAA.

Finally, the last path identifies chimeric fusion transcripts through ChimeraScan [22], a tool based on Bowtie [23] alignments to detect putative fusion breakpoints.

At present RAP is implemented for several organisms: *Homo sapiens *(genomes hg18 and hg19), *Mus musculus *(genomes mm9 and mm10), *Rattus norvegicus *(genome rn4), *Drosophila melanogaster *(genome dm3), *Saccharomyces cerevisiae *(genome sacCer3) and *Zea mays *(genomes maize2, maize3 and Mo17_v1). Additional genomes will be considered and implemented in the future, if required by users.

### Quality checks

An effective quality check is critical for a reliable data analysis, since the read quality may affect downstream results. FastQC [15] (Figure 1, step 1) provides a range of quality checking modules covering different aspects of raw read quality, helpful to highlight sequencing biases and contaminants.

NGS QC Toolkit [16] (Figure 1, step 2) is a suite of tools for quality check and filtering of NGS data from Illumina and Roche 454 platforms. This tool allows to filter out low quality reads and increase the overall dataset quality.

High quality reads are processed to extract several information at different analysis stages.

### Read mapping

Since RAP is specifically designed to work with eukaryotic genomes and to detect novel splicing events, a spliced aligner is required to map reads to the genome, across the introns. TopHat2 [17] (Figure 1, step 3), using Bowtie2 [24], can map reads against both genome and transcriptome (when an annotation is provided). Since full-length transcripts bring to a significant gain in both sensitivity and accuracy (e.g. for the right recognition of pseudogenes), RAP automatically includes transcript annotations into the pipeline. Reads are first mapped to known transcripts and unmapped reads (deriving from unknown transcripts or containing many miscalled bases) or poorly aligned reads are then aligned to the genome.

### Transcripts reconstruction and quantification

After TopHat2 execution, the resulting alignment files are provided to Cufflinks [18] (Figure 1, step 4) to generate a transcriptome assembly and to estimate the expression level of all detected isoforms. Furthermore a gene expression raw-count is also calculated with HTSeq [20].

Since RAP does not allow de novo transcript assembly, Cufflinks is guided by a gene reference annotation file (the same used during the alignment). The user can select between two reconstruction algorithms provided by Cufflinks assembler. A first option is to use reference transcripts to reconstruct known isoforms and avoid the assembly of putative novel transcripts (basic assembler). A second option is to use the supplied reference annotation to guide the assembly and include in the output both reference transcripts and novel assembled genes and isoforms (RABT assembler) [25].

Reconstructed transcripts are then analyzed to estimate their relative abundance, measured in FPKM (expected Fragments Per Kilobase of transcript per Million fragments sequenced).

RNA-Seq experiments suffer of known sequencing bias, that can jeopardize the assumption of uniform coverage. A bias can be typically introduced by the cDNA amplification phase when random hexamers are generally used [26]. Cufflinks can be used to reduce biases adopting two strategies. It estimates approximated transcripts abundance to weight reads and profiling the transcripts sequences, then abundances are re-estimated adjusting the initial approximation on the bases of detected sequencing biases (fragment bias correction). Furthermore, reads mapped to multiple genome positions are at first uniformly assigned to each position and then expression levels are re-estimated by probabilistically dividing reads on the basis of the first abundance estimation, the inferred fragment length and the fragment bias (multi reads correction).

Since most of the tools for differential gene expression analysis require raw counts, RAP also includes a second approach to estimate the relative gene expression by using the HTSeq suite [20] (Figure 1, step 5). Since reads can overlap, even partially, two or more features (e.g. exons), RAP implemented the *intersection_nonempty *mode provided by HTSeq to guarantee the highest number of assignments. With this mode if a read is completely mapped on a feature and partially on another, the read is assigned to the first feature. Furthermore, this mode is able to handle partial overlap on a single feature.

### Splice junctions detection

High quality reads, obtained from NGS QC Toolkit, are also analyzed to detect splicing junctions. This phase is executed in parallel with the previous steps. Although TopHat2 is already able to detect both known and novel splicing junctions, the algorithm implemented in this phase is more focused on this task and can detect a greater number of splicing events.

To reduce the computational load, unspliced reads, detected by Bowtie mapping to the reference genome, are discarded from the initial dataset (Figure 1, step 6). Because Bowtie2 [23] is an unspliced aligner, only intra-exonic reads will be mapped in this phase and discarded. Unmapped reads may potentially present a spliced alignment on the genome easily detectable, again using Bowtie, by mapping to a custom built splice junctions library (Figure 1, step 7).

The splice junctions library is built starting from a gene annotation model in GTF format (http://www.ensembl.org/info/website/upload/gff.html), the same already used during the alignment and the assembly steps) and includes two different categories of splice junctions: known and novel. Known junctions are directly derived from RefSeq [27] while novel junctions are obtained through a combinatorial exon skipping procedure by considering all compatible exon skipping patterns. In a transcript with *k *exons, *k-1 *known splice junctions can be observed. By selectively skipping the inner exons, *k-2 *novel splice junctions can be identified. Multiple sequential inner exons can also be skipped, picking out further junctions. This combinatorial approach exhaustively lists all $\frac{\left(k-2\right)\times \left(k-1\right)}{2}$ novel compatible splice junctions and it is applied to each annotated transcript. Splice junctions already included in the known junctions set are not counted to avoid overestimation of novel junctions.

From each splice junction, the flanking exons sequences are extracted, considering the 3' end of the upstream exon and the 5' end of the downstream exon, and spliced together. The library is produced considering several boundaries lengths (50, 75, 100 and 150bp) to have an optimal fitting with the user-provided read lengths. If a flanking exon is shorter than the selected boundary length, a truncated junction is extracted. Since the two boundaries are fused together each sequence is annotated with a length of source exons, to be able to further determine the fusion point even for truncated junctions. The human RefSeq junctions library contains about 200,000 known splice sites on a total of about 2 millions combinatorial junctions included in the library.

### Polyadenylation site detection

Reads still unmapped to the genome, transcriptome and junctions are further analyzed to identify polyadenylation sites (Figure 1, step 8). PolyA tags (reads containing a stretch of A at the end of the sequence) are extracted, trimmed and aligned to the genome. Since the read may cover a short final exon, the sequence could also contain a splice junction. Therefore a spliced alignment with TopHat2 is adopted. Following the alignment, a parsing procedure is applied to annotate the concurrent occurrence of polyadenylation signal (PAS) sequences (i.e. AAUAAA, AUUAAA or less common variants). PAS are searched in order of frequency, from the most common to the rarest. Over the canonical polyadenylation signal (AAUAAA) a total of 10 variants are considered [28].

### Cassette exons identification

Even though cassette exons (i.e. exon skipping events) can be recognized by the splice junction mapping phase, RAP also handles a more specialized step focused on this and other elementary alternative splicing (AS) events such as intron retention and alternative 5' or 3' splice sites. Indeed in higher eukaryotes, exon skipping is the most common AS event, accounting nearly 40% of all events [29]. RAP adopts a statistical method for splicing events identification and quantification implemented by SpliceTrap [21] (Figure 1, step 9).

In order to reliably detect and quantify every potential exon-skipping event, SpliceTrap builds an exon-trio database (TXdb) capturing all known transcripts obtained from RefSeq annotations [27] and breaking each transcript into all possible exon trios. Each exon trio thus leads to two sequences: an inclusion isoform with all three exons and a skipping isoform with the two flanking exons only.

Reads are then aligned to the TXdb database using Bowtie and poorly covered exon trios are filtered out applying a dynamic exon-size-dependent cut-off strategy. Exon inclusion ratios are then estimated adopting a Bayesian model, given the probability of observing each fragment on a specific isoform (as function of both inclusion and exclusion isoform expression level and normalized by the isoforms lengths) [21].

The exon inclusion ratio is defined as the expression level of the inclusion isoform divided by the total expression level of both isoforms (inclusion and exclusion) derived from an exon trio.

SpliceTrap is also able to identify other splicing events, such as intron retention (IR), alternative donor (5' splice site) (AD) and alternative acceptor (3' splice site) (AA).

### Chimeric transcripts annotation

A further RAP path carries out a specific data analysis to detect chimeric transcripts (Figure 1, step 10), i.e. RNAs encoded by a fusion gene or by two different genes through a trans-splicing event. RAP integrates ChimeraScan [22], a tool based on Bowtie alignments to detected putative fusion breakpoints. ChimeraScan aligns paired-end reads to a combined genome-transcriptome reference to discard uninformative data and to estimate the insert size distribution of the library. Unmapped reads are then trimmed into smaller segments and realigned to the genome, to build a set of putative chimeric junction sequences. Potential fusion breakpoints arise from fragments that align to distinct references or distance genomic locations (according to the previously estimated insert size distribution) of the same reference. Putative chimeric junction sequences are then used as reference to realign candidate junction-spanning reads.

### Differential expression analyses

In the case the user wishes to compare two different conditions, each eventually represented by more replicates, several differential analyses can be executed by RAP (Steps A-E in Figure 1). In particular, RAP detects differentially expressed genes and transcripts by adopting two approaches: Cuffdiff2 [25] from transcript abundances determined by Cufflinks (Figure 1, step A) and DESeq [20] from raw counts calculated by HTSeq (Figure 1, step B).

Cuffdiff2 estimates the expression changes at transcript level and controls for variability across replicate libraries, modeling variability in the number of fragments generated by each transcript across replicates. Incorrect rejections of a true null hypothesis (false positives) are controlled with the Benjamini-Hochberg correction [30] for multiple testing of differential expression (false discovery rate, FDR). However, this strategy works properly when a large number of comparisons are performed. In the case of missing replicates, the variance that can only be calculated between the conditions, reducing the statistical power.

DESeq instead introduces the assumption that the variance is a sum of a raw variance term (derived from biological variability) and shot noise term (from counts uncertainty). This method allows, with strong limitations, to extend the use of DESeq to datasets without or with very few replicates, pooling together genes with similar expression levels [20]. However, such a design should be discouraged in order to improve the accuracy and to increase the biological robustness of the results.

Other differential analyses are performed to compare results obtained at other RAP steps, specifically cassette exons (Figure 1, step C), splicing junctions (Figure 1, step D) and polyadenylation sites (Figure 1, step E). Both polyadenylation sites and splicing junctions are measured as a raw count of reads mapping the specific position and are therefore suitable to be differentially analyzed adopting DESeq. On the other hand, cassette exons are identified by an inclusion ratio and are compared using a chi-square ( χ2 ) test.

After the full analysis completion, raw output files are parsed and stored into a dedicated and optimized MySQL database.

## Results and discussion

### Web interface

The RAP pipeline described above can be accessed through an interactive web-based graphical user interface (GUI). Analysis steps are implemented and distributed inside our cloud computing environment through a dispatching application and the whole execution can be launched and monitored using a common up-to-date web browser. This makes easy for anyone to perform a complete and complex RNA-Seq analysis, also without specific technical, computer or bioinformatics skills.

The web-based GUI is written in PHP: Hypertext Preprocessor (PHP) language using HyperText Markup Language (HTML) and JQuery, combined with HTML5 and CSS3 standards, to enable a better user interaction.

A personal user account is required in order to use the web interface. An account can be requested through the registration form, providing a valid academic email address and a password. The first step to submit a dataset to RAP is the creation of a new study (or project), a collection of information about a single sequencing project. A study contains one or more input files and more analyses can be run on the same input. The creation of a new study only requires little information, such as a title, a description and an access level (private, group or public). A private study can be accessed only by the owner, while a public study will be accessed by any authenticated user. With a group access level only users sharing the same institute will be enabled to access to the project.

Before starting any analysis, the user has to upload one or more input files. The upload engine offers several options. The main method is the Web Upload, which supports up to 12GB file size on 64-bit operating systems and up to 2GB on 32-bit operating systems. The user can follow the upload progress and interact with the system adding or removing files also during the transfer.

To overcome the Web Upload limitations the user can choose to upload data providing a web link (HTTP, HTTPs and FTP protocols are allowed). In this case the user can enter one or more links and the system will handle the download using an internal queue. A third option consists in the use of the Dropbox Chooser plugin.

The upload facility supports several input formats such as text-based raw sequences produced by Illumina sequencing platforms (i.e. FASTQ [31]), pre-aligned data (i.e. BAM and SAM [32]), compressed reads (i.e. SRA [33]). The user can also upload these files in a compressed archive to speed up the uploading process (several common compressed formats are managed, such as zip, tar, gzip, bz2).

At the end of the upload, the input files can be decompressed, if required, and annotated by adding metadata information (e.g. organism, tissue, cellular line). After the annotation phase, uploaded files are imported into the project and can be used to start new analyses.

### Execution and monitoring analyses

The user can select one or more files and design a new analysis providing a name, a description and optionally modifying the analysis parameters. Several parameters can be customized to tailor the analysis, even if optimal default parameters to perform a standard workflow are suggested. Analysis parameters are divided into five categories: *Common parameters, Quality check and filtering, Genome alignment, Transcript assembly and abundance estimation, Detection of polyA reads*.

The first category contains parameters common to all or many pipelines modules, such as the reference database and the Reference-GTF. A set of flags allows to enable or disable the optional steps (i.e. Junctions Search, Cassette Exons Detection, Polyadenylation Sites Search and Fusion Transcripts Identification).

With the *Quality check and filtering *parameters the user can modify the behavior of quality control and trimming module (e.g. the cut-off value for the PHRED quality score for high-quality filtering and the primer/adaptor library).

With *Genome alignment *parameters the user can personalize the mapping phase performed by TopHat2. A set of options can be used to describe the sequencing library (e.g. strand-specific libraries and paired-end mean inner distance) and to modify the alignment behavior (e.g. the maximum number of allowed multi-hits, mismatches and length of gaps).

In the *Transcript assembly and abundance estimation *section the user can allow the reconstruction of novel-transcripts (e.g. enable Novel-Transcripts option to adopt the RABT assembler to try to reconstruct novel transcripts supported by alignment data).

Finally, in the *Detection of poly(A) reads *category, the user can tune the poly(A) extraction step.

After the analysis customization, the workflow can be submitted to the queue system. The execution process is totally automated through a dispatching architecture integrated with a Torque Resource Manager.

While the pipeline is running, the user can monitor the status of each step and access to intermediate files through preview and download operations. The running status switches from "queued" to "running" to "completed". In case of execution fault, the running status is marked as error. Known issues (e.g. an unmatched number of reads between the fragments of a pair) are reported near the error status to give to the user an explanation of the problem. Other status can be used, such as skipped (if an optional branch is not executed) and internal-error (if a step violates resources constraints, e.g. the allowed execution time or memory consumption. Further details about the infringement are described by a popup).

The final results can be browsed directly through the web interface to query, filter and sort the results.

### Results browser

**Figure 2:**
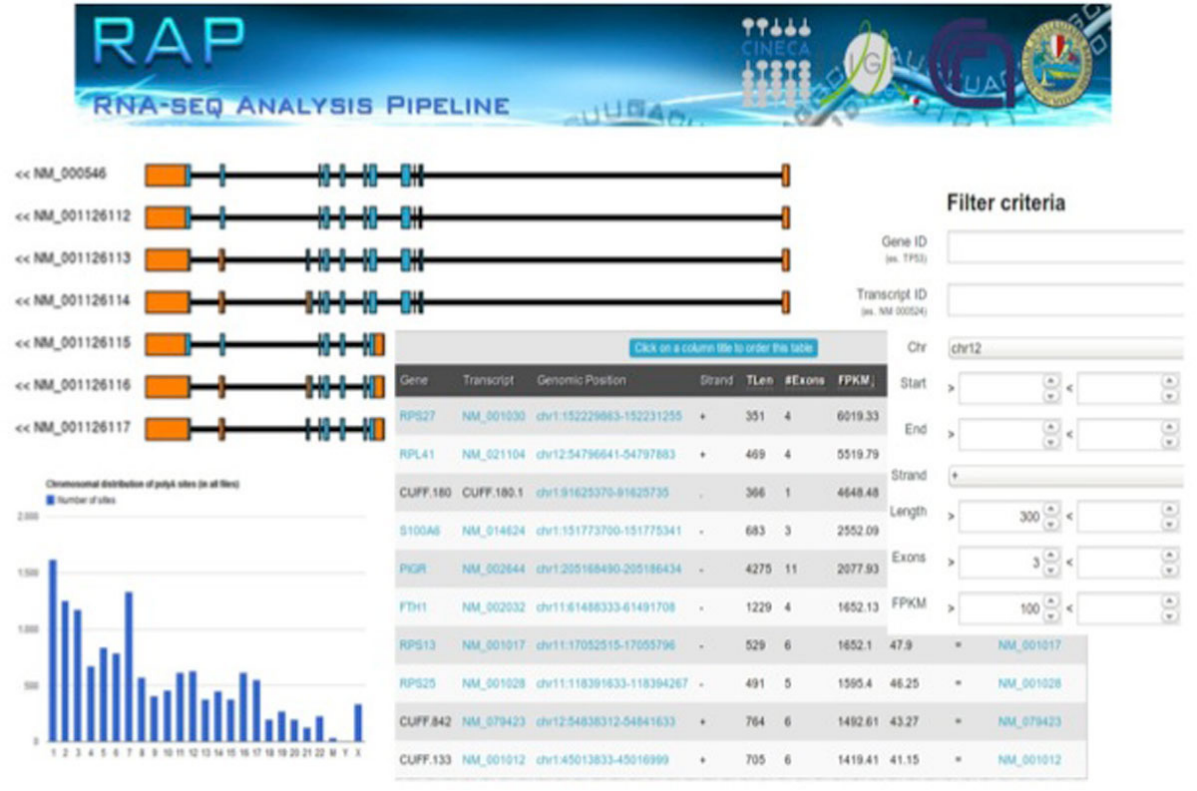
**RAP Banner and results examples**. From top left corner in counterclockwise direction: gene structure view with alternative isoforms of gene TP53, example of chromosomal distribution of polyadenylation sites, example of results table with detailed information about expressed transcripts and visualization of the query form for data filtering.

Results are organized into various sections for an easier accessibility and interpretation. Each section reports a summary of obtained results and these summaries can then be expanded to explore more detailed information (Figure 2).

Results sections are: *Quality checks, Data Summary, Gene Expression, Search by Gene, Junctions, PolyA Sites, Cassette Exons, Fusion Transcripts *and *Differential Expression*.

In the *Quality Check *section RAP organizes results obtained from both FastQC and NGS QC Toolkit in a comprehensive summary table. Color-coded labels give to the user a prompt quality overview and each label can be explored to display the corresponding output. This section reports several data metrics useful to track the analysis process by a quantitative point of view.

The *Data Summary *reports, for each input file, the total amount of short reads (both raw and high quality reads as filtered by NGS QC Toolkit), mapping metrics, junctions alignment metrics and information from poly(A) extraction phase. A coverage plot is also reported to show the average library coverage along transcripts. This section can also be used to import data (both mapping files and reconstructed transcripts) into the Integrative Genome Viewer (IGV [34]).

The *Gene Expression *section reports expression values as estimated by Cufflinks. The summary tables displays colored-boxed numbers of both expressed genes (in green) and transcripts (in blue). Each label can be clicked to open the expression overview, a detailed list of all expressed genes and transcripts in a given sample. This set of results, along with any other details page in RAP, can be filtered using customizable thresholds, to facilitate the identification of functionally significant variants. Every column can be used to filter results and filters can be combined to produce complex queries. The output tables, as reported after the application of a set of filters, can be exported as textual/excel files for offline downstream analyses.

The *Search by Gene *section allows to simultaneously query all experiment results of the analysis project. Using this form the user can retrieve the expression values of a given gene or transcript. A dynamical graphic also displays all isoforms, along with the annotated exons (as coloured boxes) and introns (as connecting line between exons).

The *Junctions *section reports results obtained by the mapping on the splice junctions library. According to the library construction, a summary table reports, for each input file, the number of RefSeq and novel junctions.

*Poly(A) Sites *Section collects results and statistics from the polyadenylation sites detection step. A chromosomal distribution of polyA tags and a frequency table of polyadenylation signals (PAS) hexamers are also shown.

The *Cassette Exons *Section provides the identified exons skipping events and other alternative splicing events (intron retention, alternative donor, alternative acceptor) annotated from each input.

Finally, the *Fusion Transcript *Section shows the identified fusion breakpoints and their chromosomal distribution.

A dedicated section allows the user to compute differential expression operations by setting up multiple combinations of comparisons at the user's choice. The user can request a specific differential expression operation by selecting desired inputs. A group must be assigned to each selected input picking out from a dropdown menu. In case of biological replicates (i.e. more inputs are available from the same sample) they can be assigned to the same group and they will be analyzed together during the differential analysis. Although at least two inputs must be selected, no upper limit is imposed and also the whole set of inputs can be included in the same differential analysis. When more than two inputs are selected, all possible pairs will be considered to call for differential expression analysis. After the selection of inputs, the user has to configure the operation parameters and specifically the type of differential expression operation: at transcript level (computed with Cuffdiff2), at gene level (computed with DESeq) or both of them.

An analogous schema is adopted to request for the determination of significant changes in cassette exons inclusion ratios, polyadenylation usage and splicing junctions.

### Case studies

RAP has been validated on several public datasets and results can be publicly accessed to any registered user.

Dillman et al. [35] used RNA-Seq to study the transcriptome of three adult (3-4 months old) female mice and four embryonic 17 days old (E17) female *C57BL/6J *mice. Authors validated 8 DE genes that showed a range of differential expression as well as different estimated expression levels in embryonic or adult tissue (*Vax1, Igf2bp1 *and *Wipf1 *as low expressed genes, *Draxin, Nrp1 *and *Caly *as moderately expressed genes and *Ttr *and *Mobp *as highly expressed genes). As negative control they selected two genes that showed low variance and were not differentially expressed (*Ppid *and *Ubc*). They also examined a group of four genes highly up-regulated in adult (*ATP10a, Grm4, Sparc, Baiap3*) and four up-regulated in embryos (*Ncapg2, Tet1, Ccnd2, Ooep*).

RAP shows a remarkable agreement with these validations (dataset name in RAP: "*Mouse cerebral cortex adult VS embryonic"*) with the only exceptions of *Igf2bp1 *and *Mobp*, both marked as non significant due to the stringency of Cuffdiff2 algorithm, although both noticeably differentially expressed.

We also compared the whole set of differential genes obtained from Dillman et al. (4125 genes) with DE genes obtained from RAP by applying the same set of filters reported by authors (4-fold or greater and p < 0.05 after FDR correction). We obtained 701 DE genes with DESeq and 611 with cuffdiff with a percentage of agreement of, respectively, 91% (636/701) and 85% (525/611). We noticed in the Dillman DE dataset a number of genes with an absolute fold change lower than 4 (in contrast to what is published in their paper) and we then proceeded to re-filter their dataset obtaining 1142 genes. Comparing RAP results with this new dataset we obtained a percentage of agreement of 81% (572/701) (DESeq) and 73% (452/611) (Cuffdiff).

Edgren et al. [36] identified 24 novel and 3 previously known fusion genes in breast cancer cells using paired-end RNA-seq. They isolated total RNA from four breast cancer cell lines (*BT-474, KPL-4, MCF-7 *and *SK-BR-3*) and sequenced using the 1G Illumina Genome Analyzer 2X (Illumina). They validated 27 fusion genes: 11 in *BT-474*, 10 in *SK-BR-3*, 3 in *KPL-4 *and 3 in *MCF-7*. A pooled dataset (obtained merging 8 lanes, two from each cellular line, and producing a single paired-end input) has been analyzed (dataset name in RAP: "*Edgren"*) and our pipeline found 8 fusions from *BT-474*, 8 from *SK-BR-3*, 3 from *KPL-4 *and 2 from *MCF-7*.

Since the merging process can create artifacts, two *BT-474 *lanes have been analyzed separately. In both of them, 10 fusions (on a total of 11) have been observed. Although *RPS6KB1-TMEM49 *fusion has not been observed, RAP reported a fusion breakpoint between *RPS6KB1 *and *SNF8*, a nearby gene known to be a partner of *RPS6KB1 *[37].

We also applied the RAP workflow to dataset from Burge et al. [3], Illumina's Human Body 2.0 [38] and Pickrell et al. [39] and results are available upon request.

## Conclusions

RNA-Seq can be profitably used to understand and quantify the complexity of eukaryotic transcriptomes, in order to investigate gene expression from different perspectives. However, the analysis of RNA-Seq data can be challenging both for the broad analysis scope and the large computational and storage resources required. The development of highly automated pipelines for data analysis is therefore critical, also to speed up research and publication.

A whole RNA-Seq analysis pipeline (RAP) has been implemented to investigate RNA-Seq data from many points of view. This pipeline performs a complete analysis to determine and quantify both genes and transcripts expression, exploit the alternative splicing identifying expressed splice junctions, poly(A) sites, cassette exons and other splicing events. Furthermore, it can also be used to investigate tumor tissues by detecting chimeric transcripts. Several differential analyses allow to compare data from many samples, determining significant changes among experimental conditions.

The execution of this pipeline has been fully automated and integrated with in house computational servers used via cloud computing.

Taking advantage of the modular structure of RAP we are considering and implementing several improvements to RAP analysis workflow, to enhance the identification and annotation of splicing junctions, novel transcripts and fusion events. We are also implementing additional downstream operations such as Pathway enrichment analysis for differentially expressed genes, identification of Allele specific expression through the integration of RAP with WEP [40] a pipeline we previously devised for whole-exome analysis, and to provide additional result data plots, both helpful for a more effective interpretation of results.

To offer to the user a wider range of tools we are considering the introduction of alternative mapping (e.g. STAR [41]), detection of differential alternative splicing analysis (e.g. MATS [42]) and expression levels (e.g. edgeR [43], NOISeq [44], baySeq [45]). Further improvements could be obtained by integrating wider annotation data obtained from ASPicDB [46], a database of reliable annotations of the alternative splicing pattern obtained by ASPic/PINTRON algorithm [47].

## Availability and requirements

RAP is freely available to academic users at http://bioinformatics.cineca.it/rap/. Each registered user can create up to 2 projects, which can contain a maximum of 2 analyses. Each project can contain up to 12 files (single or paired-end). Every lane should not exceed 15 Gb in size. Our retention policy is to keep all data stored for 30 days since the analysis has been completed.

For a more extensive use of RAP please contact hpc-service-bio@cineca.it to arrange a specific agreement with CINECA.

## List of used abbreviations

NGS: Next Generation Sequencing; RNA-Seq: RNA Sequencing; RAP: RNA-Seq Analysis Pipeline; SRA: Sequence Read Archive; BAM: Binary Alignment Map; GTF: Gene Transfer Format; PAS: PolyAdenylation Signal; AS: Alternative Splicing; TXdb: SpliceTrap exon-trio database; IR: Intron Retention; AD: Alternative Donor; AA: Alternative Acceptor; GUI: Graphical User Interface; PHP: Hypertext Preprocessor; HTML: HyperText Markup Language; CSS: Cascading Style Sheets; HTTP: Hypertext Transfer Protocol; HTTPs: HyperText Transfer Protocol over Secure Socket Layer; FTP: File Transfer Protocol; IGV: Integrative Genome Viewer; DE: Differential Expression.

## Competing interests

The authors declare that they have no competing interests.

## Authors' contributions

MD, PDDM, MP developed the web interface along with the whole analysis pipeline. GP, TC supervised the project development. GP, EP acted as scientific supervisors. GP, EP, AMDE, RC contributed to design and to validate the pipeline. MD, GP, MP, PDDM wrote the manuscript. All authors read and approved the final manuscript.

## Declarations

We acknowledge support of the publication fee by Epigen project.

This article has been published as part of *BMC Genomics *Volume 16 Supplement 6, 2015: Proceedings of the Italian Society of Bioinformatics (BITS): Annual Meeting 2014: Genomics. The full contents of the supplement are available online at http://www.biomedcentral.com/bmcgenomics/supplements/16/S6.
